# Increased resistance in splenectomized mice to a methylcholanthrene-induced tumour.

**DOI:** 10.1038/bjc.1977.118

**Published:** 1977-06

**Authors:** R. W. Chang, J. L. Turk

## Abstract

Prior splenectomy increased the resistance of BALB/c mice to a syngeneic methylcholanthrene-induced ascitic tumour inoculated i.p. The survival rate of splenectomized mice was 81-6% while those of normal and sham-operated controls were 11-5% and 20% respectively. The effect of splenectomy, however, was seen only within the dose range of 10(3) to 10(4) tumour cells. This effect of splenectomy was abolished by the transfer to mice of serum from tumour-bearing mice, and of spleen cells from normal donors, immediately after the inoculation of tumour cells. Cell-free ascitis fluid did not abolish the effect of splenectomy. The findings suggest that there is a subpopulation of spleen cells which produces a tumour growth enhancing factor which is found in the serum of tumour-bearing mice.


*Br. J. Cancer* (1977) **35**, 768.

### INCREASED RESISTANCE IN SPLENECTOMIZED MICE TO A METHYLCHOLANTHRENE-INDUCED TUMOUR

R. W. S. CHANG AND J. L. TURK

*From the Department of Pathology, Royal College of Surgeons of England, Lincoln's Inn Fields, London WC2A 3PN*

Received 10 December 1976 Accepted 18 January 1977

**Summary.—Prior splenectomy increased the resistance of BALB/c mice to a syngeneic methylcholanthrene-induced ascitic tumour inoculated i.p. The survival rate of splenectomized mice was 81·6% while those of normal and sham-operated controls were 11·5% and 20% respectively. The effect of splenectomy, however, was seen only within the dose range of $10^3$ to $10^4$ tumour cells. This effect of splenectomy was abolished by the transfer to mice of serum from tumour-bearing mice, and of spleen cells from normal donors, immediately after the inoculation of tumour cells. Cell-free ascitic fluid did not abolish the effect of splenectomy. The findings suggest that there is a subpopulation of spleen cells which produces a tumour growth enhancing factor which is found in the serum of tumour-bearing mice.**

IN recent years, various investigators have shown a renewed interest in the role of the spleen in the immune response to tumours, as the spleen is a relatively rare site for metastases when compared to the rest of the reticulo-endothelial system (Willis, 1973). *In vivo* experiments to examine the effect of splenectomy on the behaviour of tumours used several different criteria. Thus various workers have examined the effect of splenectomy on (a) the incidence of spontaneously arising tumours (Whitmore, Salerno and Rabstein, 1972; Check *et al.*, 1974); (b) the induction of tumours by chemical carcinogens (Cohen, Headley and Bryan, 1973; Akamatsu, 1975); (c) the incidence of spontaneous regression of tumours (Pollack, 1971; Ferrer and Mihich, 1968); and (d) the behaviour of transplanted tumours (Moller, 1965; Bansal and Sjogren, 1973, 1974). The results of these investigations suggest that although splenectomy has no effect on the incidence of spontaneous tumours, or on the induction of tumours by chemical carcinogens, it reduces the rate of growth of transplanted tumours, and may increase the incidence of spontaneous regression of tumours. However, several criticisms may be made of these reports. The behaviour of transplanted tumours has been followed by measurement of the size of the tumour nodule, a parameter whose significance is difficult to interpret. Furthermore, the effect of splenectomy on the survival of animals with transplanted tumours has not been reported, or has found to be negligible.

Using survival as the criterion, we present evidence here that prior splenectomy increases the host resistance to a transplanted tumour. We have also followed the behaviour of the transplanted tumour, by determining the rate of ascites formation and also by tumour cell counts.

### MATERIAL AND METHODS

*Animals.*—Inbred BALB/c mice, 7–14 weeks old, obtained from the animal house of the Institute of Basic Medical Sciences, London, were used. They were kept in plastic cages, 10 animals to each cage, and given water and food pellets (FFG(M), Dixon

Diets for Scientific Research Animals) *ad libitum*. Groups of 10 male mice were used for all experiments unless otherwise stated.

*Splenectomy.*—Seven- to 12-week-old BALB/c mice were anaesthetized with ether. Under aseptic conditions, the spleen was exposed through a left flank incision, the vessels ligated and the organ removed. The abdominal cavity was closed, usually with a single suture through all layers, and dressed with Savlon barrier cream (Imperial Chemical Industries Ltd., Cheshire). Sham-operated mice underwent the same procedure, except that their spleens were not ligated or removed. Operative deaths were usually the result of an overdose of ether, and when this was avoided a mortality of less than 1% was achieved. Splenectomy was performed 2 weeks before tumour inoculation.

*Tumour.*—An ascitic form of a methylcholanthrene-induced fibrosarcoma (MCA) was used. It was originally obtained from the Sloan–Kettering Institute for Cancer Research, New York, and has been maintained in the laboratory by serial passage in male BALB/c every 2 weeks. The passage dose was $5 \times 10^4$ tumour cells, suspended in phosphate-buffered saline.

*Tumour-cell suspensions.*—The ascitic tumour was obtained by tapping the abdominal cavity of tumour-bearing animals. The tumour cells were washed twice in ice-cold phosphate-buffered saline (PBS) at 250 *g* for 10 min. No attempt was made to remove the small number of contaminating red blood cells. Viability determined by dye exclusion (0·2% of trypan blue in PBS) in the chamber of an improved Neubauer haemocytometer was usually over 90%. Cells were diluted to the required concentration with PBS and injected in 0·1-ml volumes. The i.p. route was used in all experiments.

*Spleen-cell suspensions.*—Spleens removed from mice killed by cervical dislocation were crushed gently in ice-cold PBS with a glass rod in glass centrifuge tubes. Tissue clumps were allowed to settle and the cell suspension removed. It was washed once with PBS and centrifuged at 250 *g* for 10 min. Red cells were lysed by hypotonic shock as follows: the spleen cell pellet was resuspended in 1 ml of PBS, about 4 ml of sterile water was added, and the suspension stirred with a Pasteur pipette for 5 s, when 6 ml of PBS was added. The suspension of spleen cells was then washed for 5 min and resuspended in PBS. Viability as determined by trypan blue exclusion was usually more than 80%.

*Mouse serum.*—Mice were bled by division of the thoracic part of the inferior vena cava under ether anaesthesia, and the serum was separated out by centrifugation after clotting. Serum from tumour-bearing mice was obtained from donors that had received $10^3$ MCA tumour cells i.p. 2 weeks previously.

*Cell-free ascitic fluid.*—Ascitic fluid containing tumour cells, obtained by tapping the abdominal cavities of tumour-bearing animals that had received $10^3$ tumour cells i.p. 2 weeks previously, was spun twice at 500 *g* for 10 min. The supernatant fluid was removed, and examined under the microscope to check that it was free of cells.

*Determination of survival rates and mean time of death of tumour-bearing mice.*—The fate of all test and control animals was followed for at least 50 days after tumour inoculation: some mice were kept for more than 270 days. The survival rate and mean time of death were calculated. The data presented here are usually the pooled results of several experiments.

*Rate of formation of ascites.*—All mice were individually marked and weighed on the first day of the experiment, so that the change in weight of each animal could be recorded. The average weight of each group of animals on various days was determined and expressed as a percentage of the average weight of the group on Day 0. The animals were usually weighed every 3 to 5 days, but more often when ascites formation was rapid.

A preliminary experiment was performed to check that the natural gain in weight of the animals was of an order that would not invalidate this method of estimation of the rate of formation of ascites. The rate of gain in weight of 10 mice was recorded from the day they were born until they were 22 weeks old. The gain in weight reached a near-plateau at about 7 weeks, after which the gain in weight was 2% per week. Animals with ascitic tumour gained about 30% in weight per week. The animals that succumbed to the ascitic tumour usually died before showing signs of malignant cachexia. The weight of dead animals drained of their ascitic fluid was usually within 2 g of their original weight.

*Growth rate of tumour cells.*—Normal and splenectomized mice were injected i.p. with $10^3$ tumour cells on Day 0. On various days thereafter, 5 mice from each group were killed by cervical dislocation. The abdominal cavity was opened and ascitic fluid collected with a Pasteur pipette from those mice with obvious ascites. The abdominal cavities of all mice were washed out with 5 ml of PBS. The final volumes of fluid collected was measured, the number of tumour cells per unit volume was determined on a haemocytometer, and the total number of tumour cells in the peritoneal cavity of each mouse was calculated.

### RESULTS

### 1. *Survival of splenectomized mice given different doses of tumour cells*

The survival of splenectomized mice given different doses of tumour cells was compared with those of normal and sham-operated mice (Fig. 1). There was no difference between the survival rates of the 3 groups when given more than $5 \times 10^4$ tumour cells, as all the animals died. There was a slight increase in the survival rate of the splenectomized group given $10^4$ cells: 23·8% compared with 6·7% for normal and 10% for sham-operated mice ($P < 0{\cdot}01$).

There was a marked improvement in the survival rate of splenectomized mice that received $10^3$ tumour cells, 81·6% compared with 11·5% and 20% for normal and sham-operated animals respectively. This is highly significant, with $P < 0{\cdot}001$ calculated by the $\chi^2$ test.

A rather surprising finding was that the survival rate for the splenectomized group was not much better than that for normal and sham-operated animals given $10^2$ tumour cells ($0{\cdot}1 < P < 0{\cdot}2$).

The mean time of death (MTD) of the 3 groups of animals was compared (Fig. 2). There was no difference between the MTDs of the animals that died from the tumour in all 3 groups.

### 2. *Rate of formation of ascites*

The rate of formation of ascites for each of the 3 groups was determined for mice given $10^3$ tumour cells (Fig. 3).

FIG. 1.—Survival of splenectomized mice (▲) compared with normal (□) and sham-operated controls (■).

FIG. 2.—Mean time of death of splenectomized mice (▲) compared with that of normal (□) and sham-operated controls (■).

FIG. 3.—Rate of formation of ascites by normal (□), sham-operated (■) and splenectomized mice (▲) inoculated i.p. with $10^3$ MCA tumour cells. Each curve represents the mean weight of 10 animals. The mean weight of each group at Day 0 is expressed as 100.

FIG. 4.—Growth of MCA tumour cells in splenectomized (▲) and normal mice (□) given $10^3$ tumour cells. Each point is the mean of 5 animals.

There was a rapid increase in the weight and, presumably, the amount of ascites formed, in normal and sham-operated mice from Day 12. These animals began to die when their weight reached more than 130% of their original weight. Splenectomized mice, however, gained relatively little in weight.

### 3. *Growth rate of ascites tumour cells*

The growth of MCA tumour cells within the peritoneal cavity of normal and splenectomized mice inoculated with $10^3$ tumour cells is shown in Fig. 4. Each point in the curves represents the mean number of tumour cells recovered from 5 animals. In normal mice, the number of tumour cells increased rapidly from $10^3$ cells to $10^6$ cells in the first 4 days, indicating a doubling time of $9\frac{1}{2}$ h. The number of cells continued to increase thereafter, but at a much slower rate. The doubling time for the second phase was about 38 h, which is 4 × slower than the first phase. The animals began to die from Day 16, when the number of tumour cells reached more than $10^8$.

The tumour cells multiplied at the same rate in splenectomized as in normal animals until Day 4, after which the number of tumour cells recovered decreased, until none were found after Day 20. About 10–30% of splenectomized animals succumbed to the MCA tumour. Those animals with obvious external signs of ascites after Day 15 were excluded from the tumour-cell count, since the point of the experiment was to follow the behaviour of tumour cells in animals protected by prior splenectomy.

The results suggest that splenectomy protected BALB/c mice from a dose of

$10^3$ tumour cells, a dose which killed over 80% of normal mice. The protective effect was apparently associated with an event manifesting itself about 4 days after tumour inoculation. Those animals that died did so at about the same time as normal or sham-operated animals.

*4. The effect of transferring spleen cells to splenectomized mice*

Since splenectomy protected mice given $10^3$ MCA tumour cells i.p., the possibility that the effect would be reversed by the injection of spleen cells was considered. So $10^8$ spleen cells from normal syngeneic mice (one spleen equivalent) were injected i.p. into a group of mice immediately after $10^3$ tumour cells had been given by the same route. A similar experiment was done with $10^8$ spleen cells taken from mice that had received $5 \times 10^4$ tumour cells 10 days previously.

Spleen cells from both normal and tumour-bearing donors reversed the protective effect of prior splenectomy (Fig. 5). Those animals, however, that received spleen cells from tumour-bearing animals died earlier (MTD = 12·5 days) than animals given the same number of normal spleen cells (MTD = 18·2 days). The presence of metastatic tumour cells in the spleens from tumour-bearing donors, increasing the effective tumour dose, was considered to be a likely explanation for this observation. This was confirmed by another experiment, in which it was found that normal mice developed the ascitic tumour 12 days after receiving $10^8$ spleen cells from tumour-bearing donors.

*5. The effect of transferring serum from tumour-bearing mice*

The presence in serum from tumour-bearing mice of blocking factors which enhance the growth of tumour has been reported by many workers (Hellstrom, Hellstrom and Sjogren, 1970; Sjogren *et al.*, 1971). So the possibility that the effect of splenectomy may be reversed by the transfer of serum from tumour-bearing mice was tested. Serum obtained from tumour-bearing mice was diluted 1/2 and used immediately; 0·1-ml volumes were given i.p. to splenectomized mice that had just received $10^3$ tumour cells by the same route.

One control group of mice received 0·1-ml volumes of normal serum similarly diluted 1/2. A second group of splenectomized animals received tumour cells alone.

FIG. 5.—The effect of transfer of $10^8$ spleen cells to splenectomized mice given $10^3$ tumour cells on the same day. SpX = splenectomy; SpN = Normal spleen cells; SpT = spleen cells from tumour-bearing animals; N = normal mice. At least 10 animals in each group.

FIG. 6.—Effect of transferring serum or ascitic fluid on the survival of splenectomized mice injected with $10^3$ tumour cells. 10 animals in each group.

Since it was possible that ascitic fluid from tumour-bearing animals might also contain blocking factors, a fourth group of splenectomized animals was given cell-free ascitic fluid in addition to tumour cells.

Serum from tumour-bearing mice reversed the protective effect of splenectomy (Fig. 6). Normal serum and cell-free ascitic fluid had no effect.

### DISCUSSION

The most striking finding of this study is that splenectomized BALB/c mice were protected from death caused by the growth of a methylcholanthrene-induced ascitic tumour inoculated i.p. The degree of protection was marked: 81·6% of splenectomized mice survived a tumour dose that killed 88·5% of normal and 80% of sham-operated controls, a 4-fold improvement in the survival rate. While these results show increased resistance as a short-term effect of splenectomy, preliminary experiments indicate that this effect may be seen up to 5 months after splenectomy.

The protective effect of splenectomy was abolished by the transfer of normal spleen cells. Further it was found that, while $10^8$ normal spleen cells (1 spleen equivalent) were able to abolish the protective effect of splenectomy, $10^6$ and $10^7$ normal spleen cells did this only partially and $10^5$ normal spleen cells had no effect.

Previous reports used the size of tumour nodule to monitor the effect of splenectomy, and the ultimate survival of the animals was not noted. This study shows a protective effect of splenectomy using survival as the criterion. With this criterion, it was possible to obtain a more clear-cut end point—the animals either died or lived. Further, there was good correlation with the rate of ascites formation and the tumour cell counts. When tumour nodule size is measured, it is never clear what is being measured, as there may be necrotic areas present and also cellular infiltrate. This study therefore provides unequivocal evidence of increased resistance to transplanted tumours in splenectomized mice.

The finding that normal spleen cells abolished the protective effect of splenectomy indicates that the anatomical integrity of the spleen is not a prerequisite for the sensitization of the cells that are responsible for the enhancement of tumour growth. This is not surprising, since it is well known that incubation of spleen cells with tumour cells *in vitro* results in their becoming sensitized to tumour antigens.

The protective effect of splenectomy was seen only within a fairly narrow range of tumour cell dose: $10^3$ to $10^4$ cells. This suggests that within this dose range, the host–tumour relationship is finely balanced and that it may be tipped in favour of the host if the tumour-growth-enhancing cells are removed by splenectomy.

A possible implication of the present observation is that incidental splenectomy during the surgical resection of carcinoma of the stomach or colon may affect the long term prognosis in these patients. It would be of interest to conduct a survey to see if this is the case.

The model described in this investigation demonstrated a protective effect of splenectomy performed *before* the establishment of the tumour, a procedure which could not be used therapeutically. Therefore one of the most obvious lines for future investigation would be to see if splenectomy performed *after* the introduction of a tumour can protect the host.

A second observation concerning the dose-dependent effect of splenectomy was that there was little difference among the survival rates of splenectomized, normal and sham-operated mice given $10^2$ tumour cells. Norlund and Gershon (1975) reported a similar finding. They transplanted a syngeneic murine melanoma (Cloudman S91) to DBA/2 mice, and found that tumours appeared earlier in splenectomized animals than in normal animals when a small tumour inoculum ($10^4$ cells) was used. The converse happened with a larger tumour inoculum ($10^6$ cells). They suggested that the immunosuppressive effect of the spleen with larger amounts of antigen was due to the stimulation of suppressor T cells. They offered no explanation for the immunopotentiating role of the spleen with smaller amounts of antigen. Perhaps splenic suppressor T cells or enhancing antibody-producing cells are not stimulated by a small antigenic load.

Tumour cells in both normal and splenectomized mice divided rapidly during the first 4 days after tumour inoculation, cell doubling time being about 9½ h. After Day 4, although the rate of division of the tumour cells in normal animals was reduced, they continued to multiply until the animals died with $10^8$ to $10^9$ tumour cells in their peritoneal cavities from Day 16 onwards, but in splenectomized animals, the number of tumour cells began to fall about Day 4 and continued to disappear until Day 18, when the animals were free of tumour cells.

The following hypothesis is proposed to explain this phenomenon, and represented diagrammatically in Fig. 7. It is suggested that the slope of the line AB represents the rate of division of tumour cells in both normal and splenectomized animals before an immune response has been established, a rate that would have continued along BE if there was no

FIG. 7. Diagrammatic representation of tumour cell kinetics in normal and splenectomized animals. For explanation see Discussion.

response. The slope of the line BC represents the net rate of killing of tumour cells in splenectomized hosts by sensitized killer cells, unhampered by suppressor cells or blocking factors that had previously been removed by splenectomy. BD represents the net growth rate of tumour cells, which allows for both the killing of tumour cells by effector cells and the greater enhancement of tumour growth by either splenic suppressor cells and/or blocking factors which inhibit the effector cells.

Serum from tumour-bearing animals abolished the protective effect of splenectomy. Biddle (1976) has recently reported similar findings using B1 × C3H mice and a syngeneic methylcholanthrene-induced tumour. He showed that serum from tumour-bearing mice, when mixed with specific tumour cells before inoculation into syngeneic recipients that had been crippled immunologically by adult thymectomy and whole-body irradiation, was able to enhance the growth of transplanted tumour, as measured by the size of the subcutaneous nodule 6 weeks later. This enhancing effect was absorbed by incubation with specific tumour cells.

There have been many reports documenting the presence of blocking factors in sera from tumour-bearing animals. Hellstrom *et al.* (1970) reported that serum from tumour-bearing BALB/c mice inhibited the cellular response of lymph-node cells to a syngeneic methylcholanthrene-induced tumour, as demonstrated by the colony inhibition assay. They also found that there was less blocking activity in serum obtained from tumour-bearing mice that had been splenectomized. Sjogren *et al.* (1971) provided evidence that suggested that the blocking factors were antigen–antibody complexes. Bowen, Robins and Baldwin (1975) also demonstrated the presence of blocking antigen–antibody complexes in serum obtained from rats bearing the rat hepatoma D23.

The inability of cell-free ascitic fluid to reverse the protective effect of splenectomy was unexpected, as it was thought to be a likely source of blocking factors. However, it is possible that since ascitic fluid bathes millions of tumour cells in the peritoneal cavity of the tumour-bearing mice, the blocking factors will have adsorbed to the surface of those cells. The adsorption of serum blocking factors by incubation with specific tumour cells has recently been reported (Biddle, 1976).

Our findings therefore suggests that there is a subpopulation of spleen cells which produces a tumour-growth-enhancing factor that is found in the serum of tumour-bearing mice. The characterization of the cell population involved will be the subject of a further communication. In addition it is planned to characterize the serum factor, particularly with respect to its specificity and chemical relation to immunoglobulin.

This work was supported by a Fellowship from the Bernard Sunley Charitable Foundation and also by funds from the Cancer Research Campaign. The authors would also like to thank Dr Nina Wedderburn for her criticisms and suggestions.

### REFERENCES

AKAMATSU, Y. (1975) Neoplasma in Strains of Splenectomized Mice after a Single 7,12-Dimethylbenz(a)anthracene Treatment. *J. natn. Cancer Inst.*, **55**, 893.

BANSAL, S. C. & SJOGREN, H. O. (1973) Correlation between Changes in Antitumour Immune Parameters and Tumour Growth *In vivo* in Rats. *Fed. Proc.*, **32**, 165.

BANSAL, S. C. & SJOGREN, H. O. (1974) Anti-tumour Immune Response and its Manipulation in a Tumour-bearing Host. *Israel J. med. Sci.* **10**, 939.

BIDDLE, C. (1976) Stimulation of Transplanted 3-Methylcholanthrene-induced Sarcomas in Mice by Specific Immune and Normal Serum. *Int. J. Cancer*, **17**, 755.

BOWEN, J. G., ROBINS, R. A. & BALDWIN, R. W. (1975) Serum Factor Modifying Cell Mediated Immunity to Rat Hepatoma D23 Correlated with Tumour Growth. *Int. J. Cancer*, **15**, 640.

CHECK, J. H., BRADY, L. W., LEIPOLD, P. L. & O'NEILL, E. A. (1974) Protection against Transplanted and Spontaneous Lymphoma by Inoculation of Heat-altered Syngeneic Tumor Cells in Splenectomized Mice. *Cancer, N.Y.*, **34**, 197.

COHEN, S. M., HEADLEY, D. B. & BRYAN, G. T. (1973) The Effect of Adult Thymectomy and Adult Splenectomy on the Production of Leukaemia and Stomach Neoplasms in Mice by N-(4-(5-Nitro-2-furyl)-2-thiazolylacetamide. *Cancer Res.*, **33**, 637.

FERRER, J. F. & MIHICH, E. (1968) Effect of Splenectomy on the Regression of Transplantable Tumours. *Cancer Res.*, **28**, 1116.

HELLSTROM, I., HELLSTROM, K. E. & SJOGREN, H. O. (1970) Serum mediated Inhibition of Cellular Immunity to Methylcholanthrene-induced Murine Sarcomas. *Cell. Immunol.* **1**, 18.

MOLLER, E. (1965) Interaction between Tumor and Host during Progressive Neoplastic Growth in Histoincompatible Recipients. *J. natn. Cancer Inst.*, **35**, 1053.

NORLUND, J. J. & GERSHON, R. K. (1975) Splenic Regulation of the Clinical Appearance of Small Tumours. *J. Immunol.*, **114**, 1486.

POLLACK, S. B. (1971) Effect of Host Sex and Splenectomy on Molony Virus-induced Sarcomas. *Int. J. Cancer*, **8**, 264.

SJOGREN, H. O., HELLSTROM, I., BANSAL, S. C. & HELLSTROM, K. E. (1971) Suggestive Evidence that " blocking Antibodies " of Tumor-bearing Individuals may be antigen–antibody Complexes. *Proc. natn. Acad Sci. U.S.A.* **68**, 1372.

WHITMORE, C. E., SALERNO, R. A. & RABSTEIN, L. A. (1972) Effects of Thymectomy, Splenectomy and 3-Methylcholanthrene on Neoplasia Expression, Incidence and Latency in AKR Mice. *Proc. Soc. exp. Biol. Med.*, **141**, 890.

WILLIS, R. A. (1973) *The Spread of Tumour in the Human Body*. 3rd Ed. London: Butterworths.


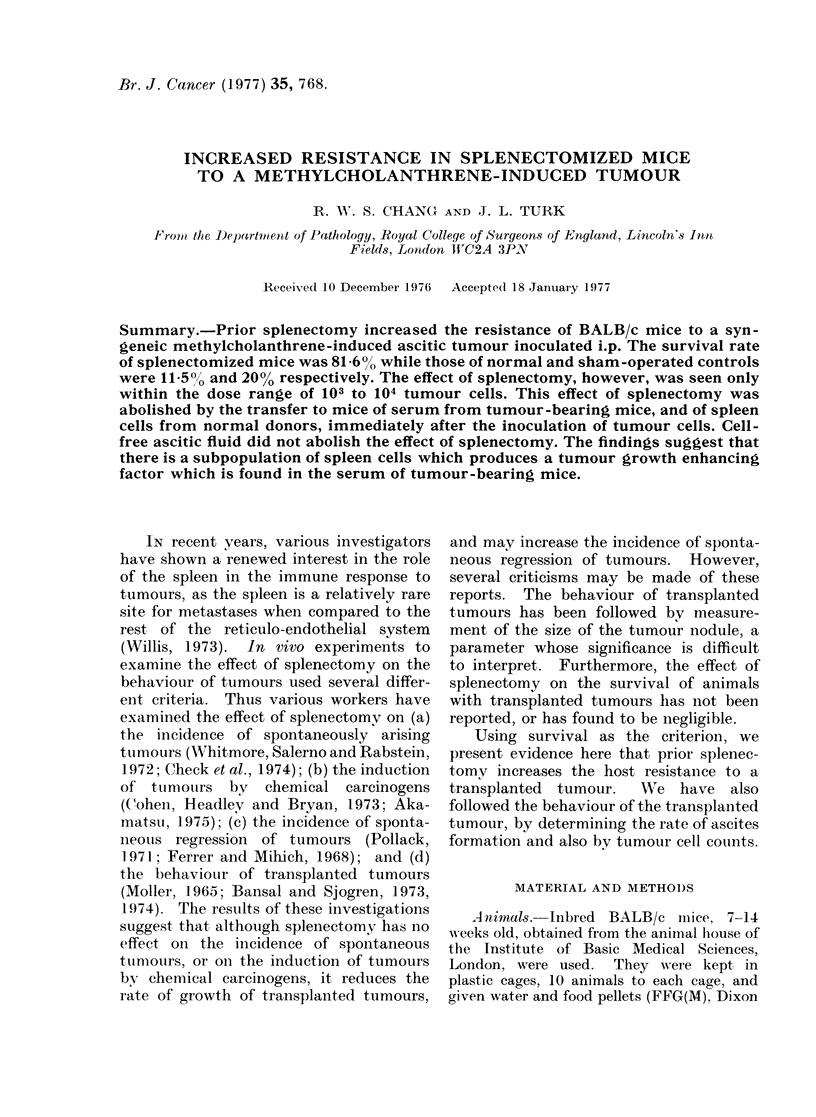

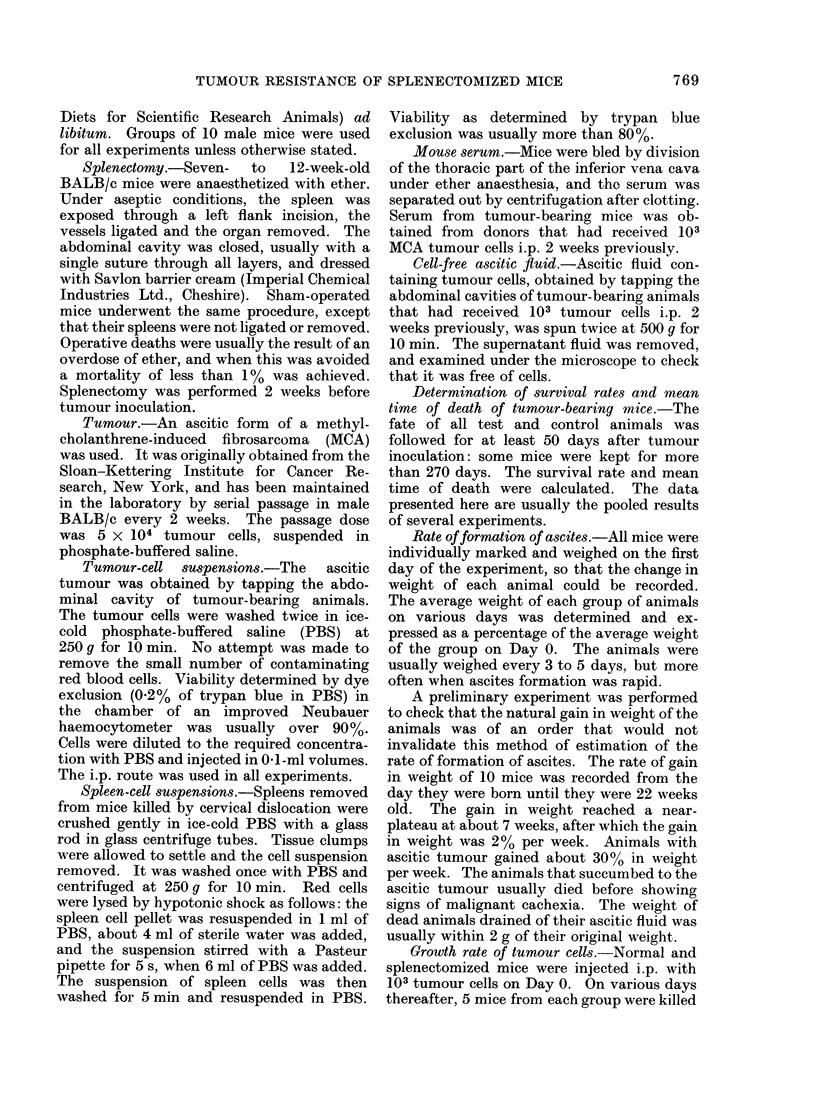

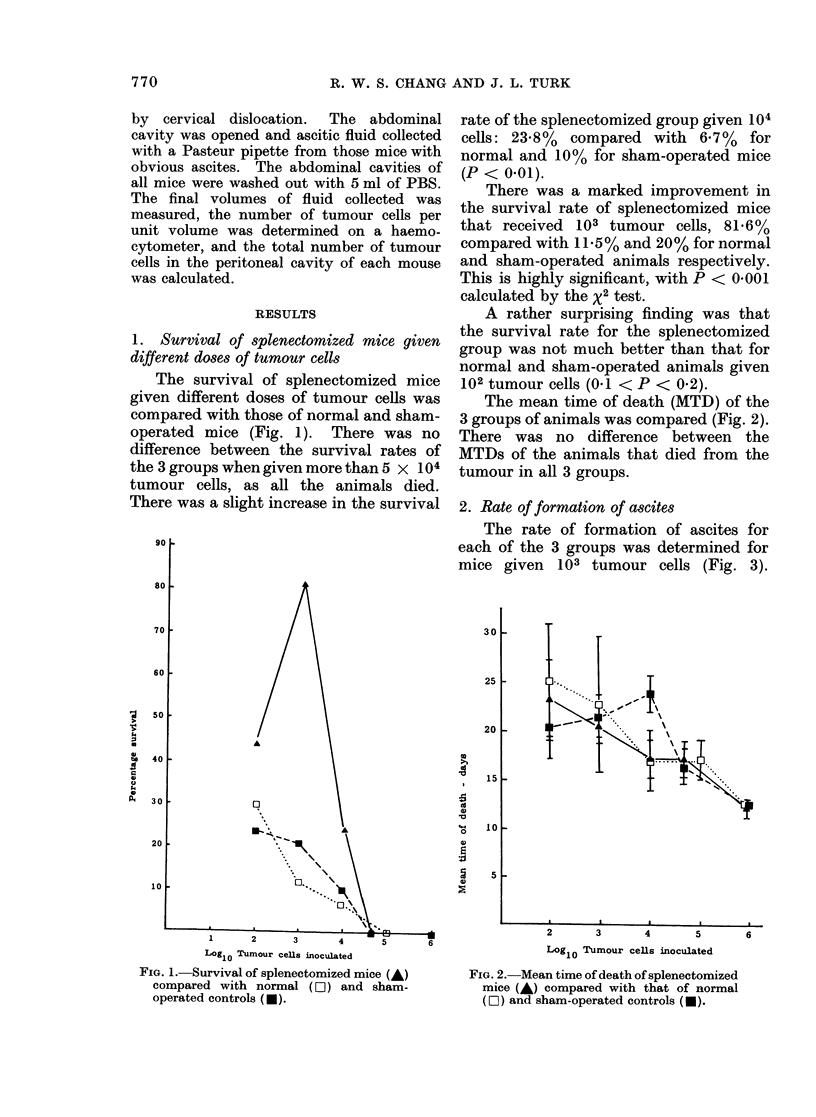

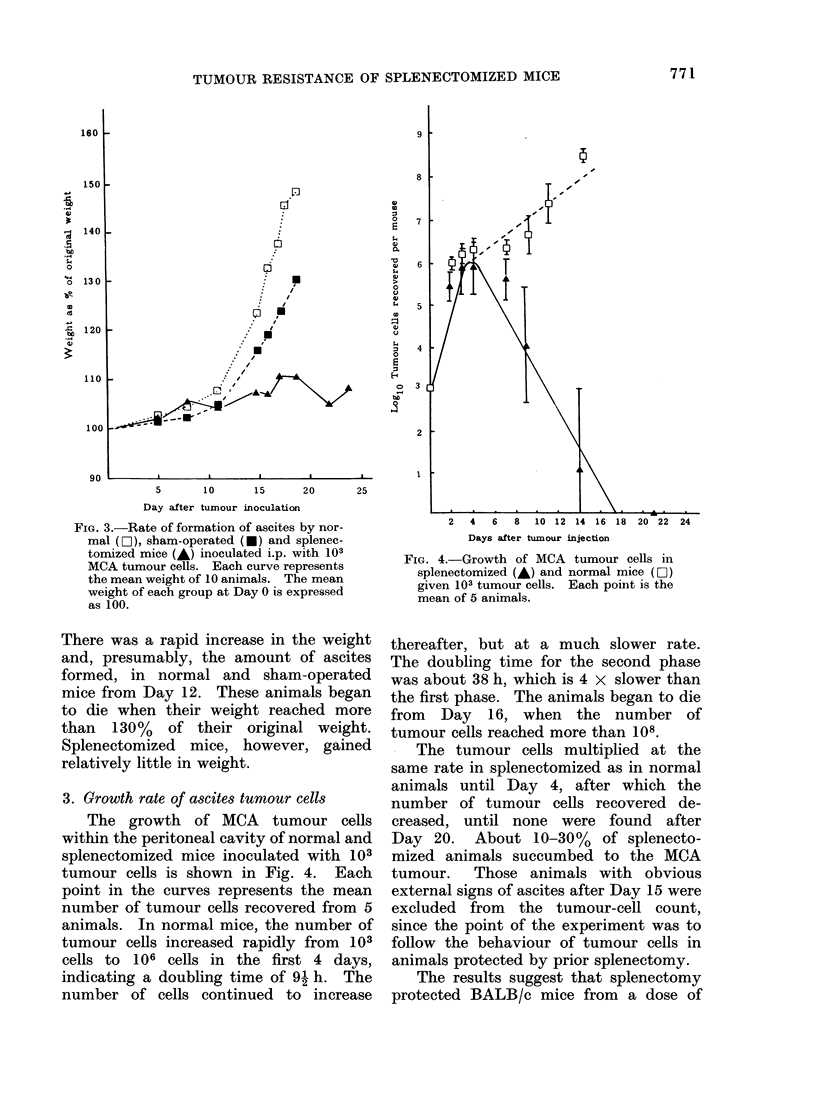

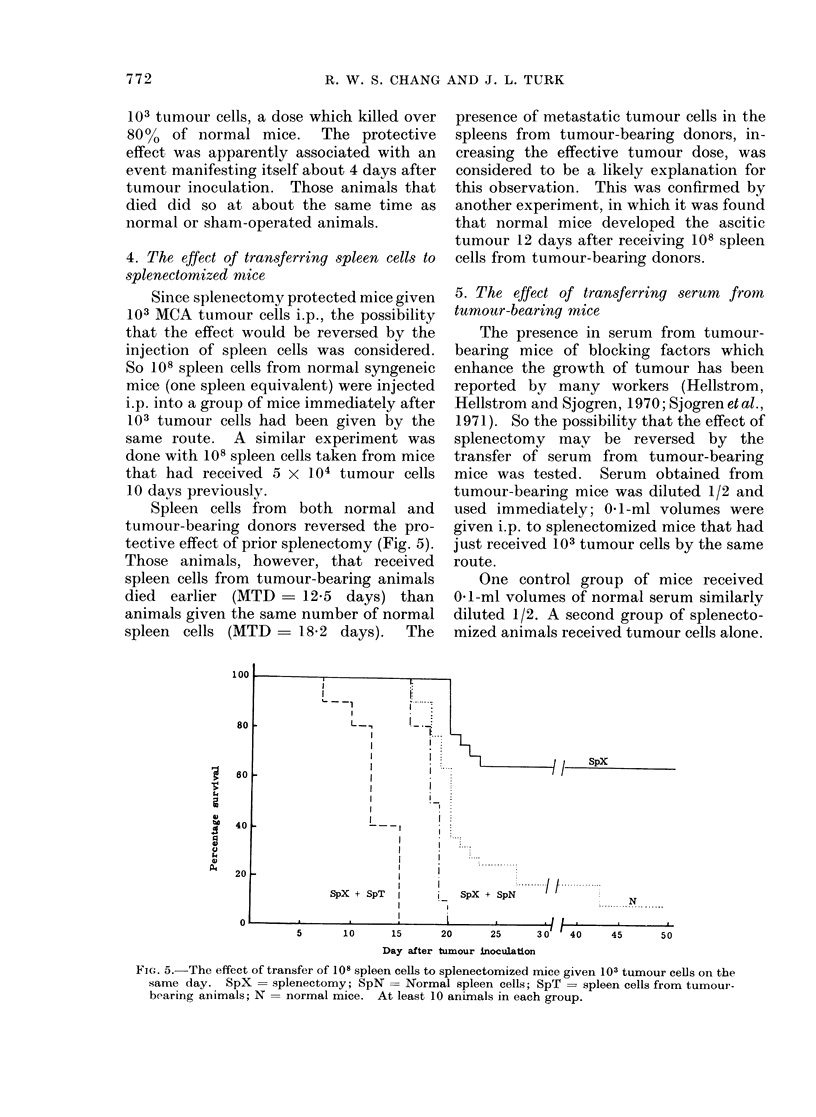

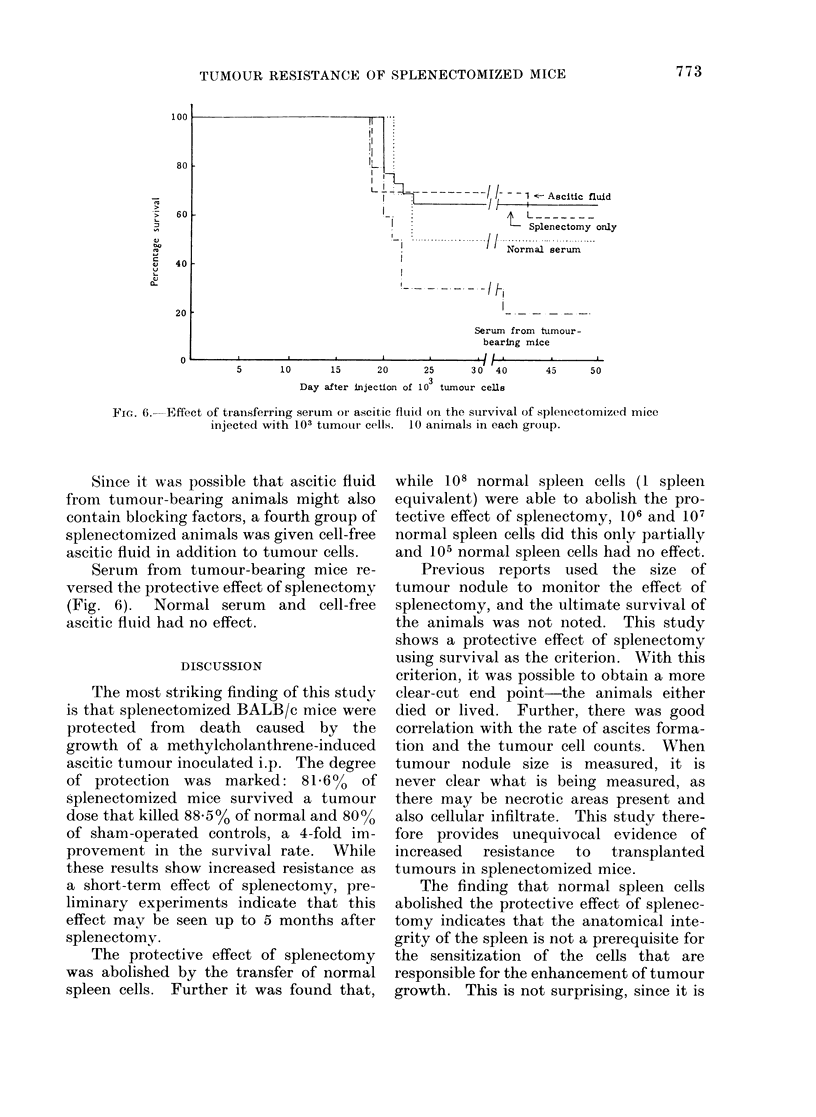

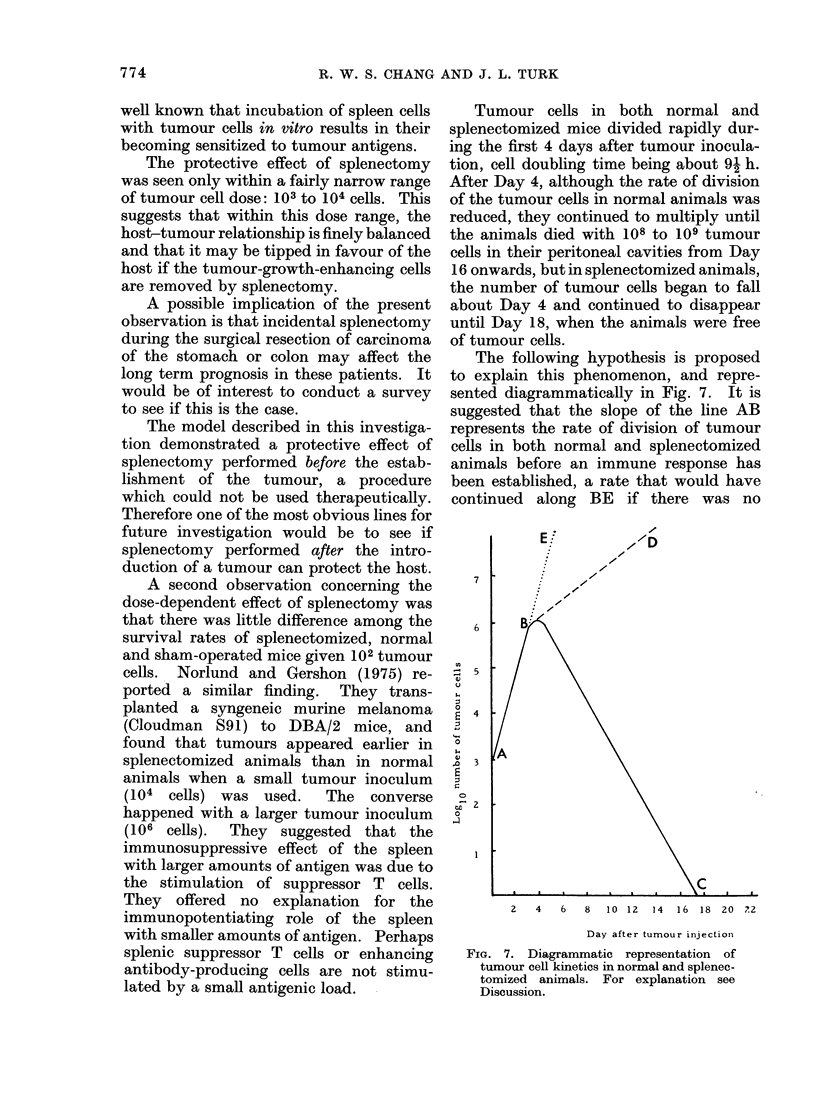

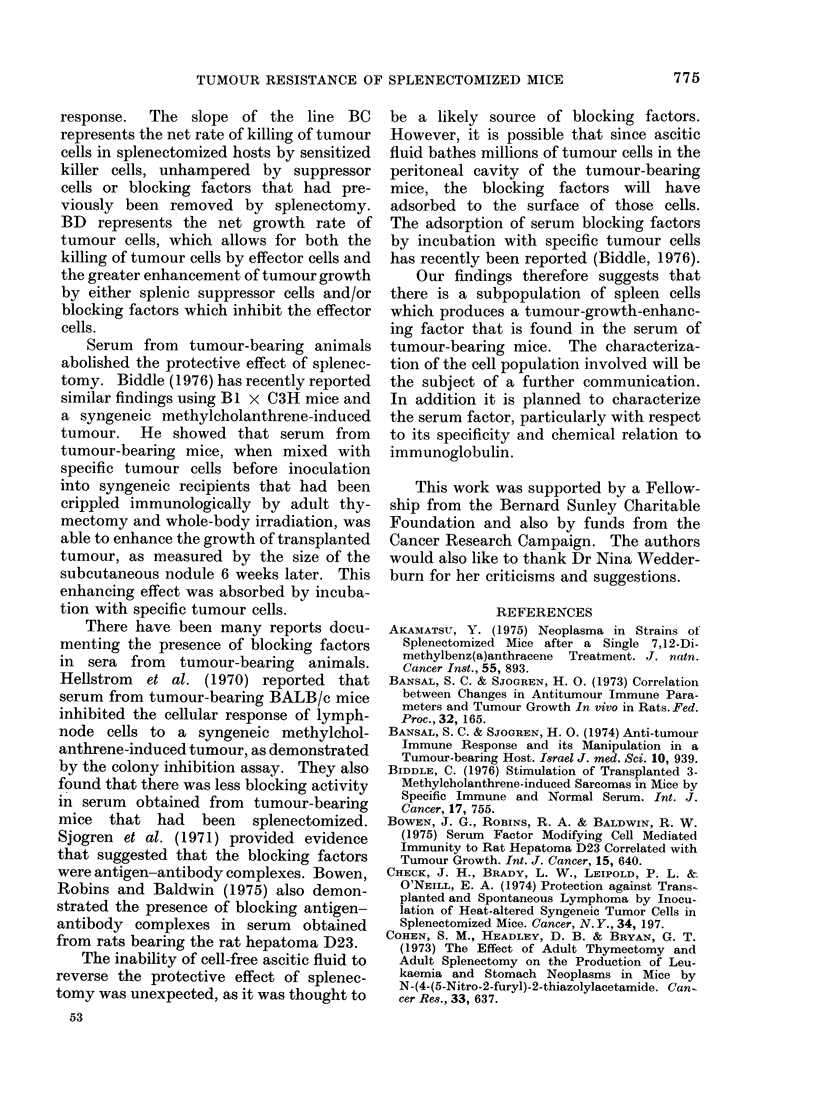

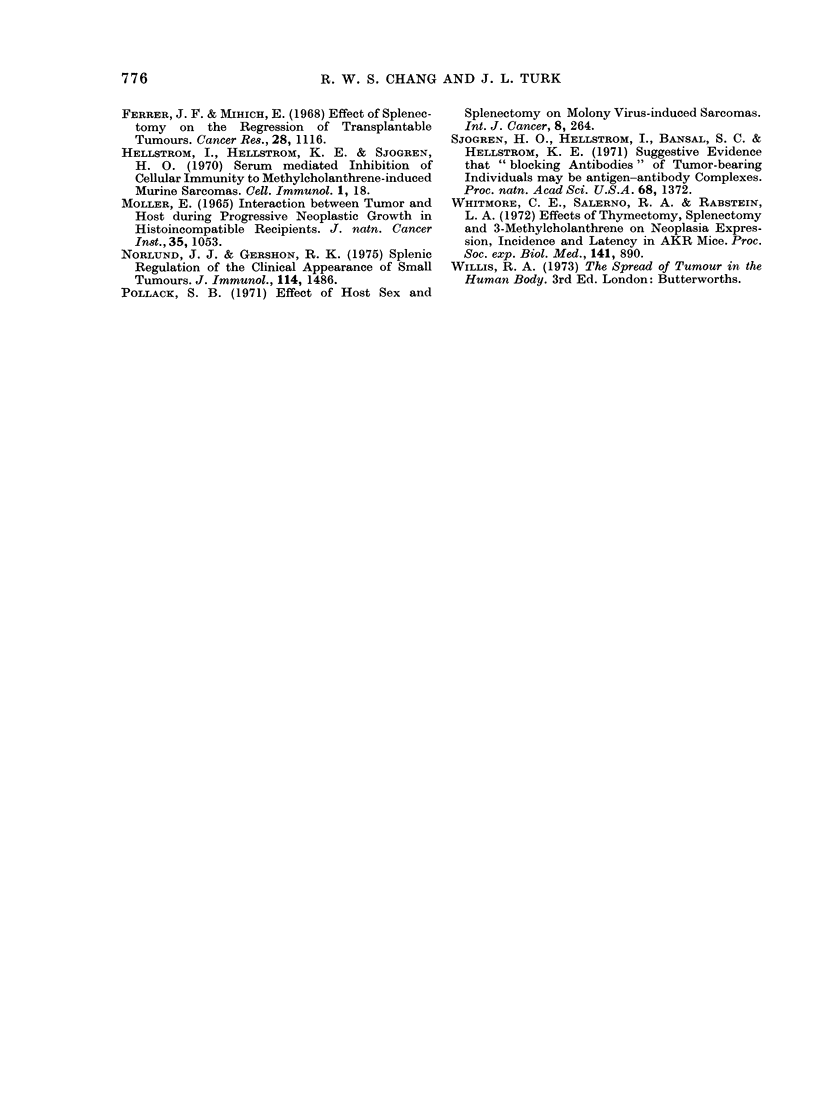


## References

[OCR_00937] Akamatsu Y. (1975). Neoplasms in strains of splenectomized mice after a single 7,12-dimethylbenz[alpha]anthracene treatment.. J Natl Cancer Inst.

[OCR_00943] Bansal S. C., Sjögren H. O. (1973). Correlation between changes in antitumor immune parameters and tumor growth in vivo in rats.. Fed Proc.

[OCR_00953] Biddle C. (1976). Stimulation of transplanted 3-methylcholanthrene-induced sarcomas in mice by specific immune ahd by normal serum.. Int J Cancer.

[OCR_00959] Bowen J. G., Robins R. A., Baldwin R. W. (1975). Serum factors modifying cell mediated immunity to rat hepatoma d23 correlated with tumour growth.. Int J Cancer.

[OCR_00965] Check J. H., Brady L. W., Leipold P. L., O'Neill E. A. (1974). Protection against transplanted and spontaneous lymphoma by inoculation of heat-altered syngeneic tumor cells in splenectomized mice.. Cancer.

[OCR_00972] Cohen S. M., Headley D. B., Bryan G. T. (1973). The effect of adult thymectomy and adult splenectomy on the production of leukemia and stomach neoplasms in mice by N-(4-(5-nitro-2-furyl)-2-thiazolyl)acetamide.. Cancer Res.

[OCR_00982] Ferrer J. F., Mihich E. (1968). Effect of splenectomy on the regression of transplantable tumors.. Cancer Res.

[OCR_00987] Hellström I., Hellström K. E., Sjögren H. O. (1970). Serum mediated inhibition of cellular immunity to methylcholanthrene-induced murine sarcomas.. Cell Immunol.

[OCR_01004] Pollack S. B. (1971). Effect of host sex and splenectomy on moloney virus-induced sarcomas.. Int J Cancer.

[OCR_01009] Sjögren H. O., Hellström I., Bansal S. C., Hellström K. E. (1971). Suggestive evidence that the "blocking antibodies" of tumor-bearing individuals may be antigen--antibody complexes.. Proc Natl Acad Sci U S A.

[OCR_01016] Whitmire C. E., Salerno R. A., Rabstein L. S. (1972). Effects of thymectomy, splenectomy and 3-methylcholanthrene on neoplasia expression, incidence and latency in AKR mice.. Proc Soc Exp Biol Med.

